# Architecture of the Entorhinal Cortex A Review of Entorhinal Anatomy in Rodents with Some Comparative Notes

**DOI:** 10.3389/fnsys.2017.00046

**Published:** 2017-06-28

**Authors:** Menno P. Witter, Thanh P. Doan, Bente Jacobsen, Eirik S. Nilssen, Shinya Ohara

**Affiliations:** ^1^Functional Neuroanatomy, KavlI Institute for Systems Neuroscience, Center for Computational Neuroscience, Egil and Pauline Braathen and Fred Kavli Center for Cortical Microcircuits, NTNU Norwegian University of Science and TechnologyTrondheim, Norway; ^2^Division of Systems Neuroscience, Tohoku University Graduate School of Life ScienceSendai, Japan

**Keywords:** parahippocampal region, hippocampus, connectivity, primate, rodent

## Abstract

The entorhinal cortex (EC) is the major input and output structure of the hippocampal formation, forming the nodal point in cortico-hippocampal circuits. Different division schemes including two or many more subdivisions have been proposed, but here we will argue that subdividing EC into two components, the lateral EC (LEC) and medial EC (MEC) might suffice to describe the functional architecture of EC. This subdivision then leads to an anatomical interpretation of the different phenotypes of LEC and MEC. First, we will briefly summarize the cytoarchitectonic differences and differences in hippocampal projection patterns on which the subdivision between LEC and MEC traditionally is based and provide a short comparative perspective. Second, we focus on main differences in cortical connectivity, leading to the conclusion that the apparent differences may well correlate with the functional differences. Cortical connectivity of MEC is features interactions with areas such as the presubiculum, parasubiculum, retrosplenial cortex (RSC) and postrhinal cortex, all areas that are considered to belong to the “spatial processing domain” of the cortex. In contrast, LEC is strongly connected with olfactory areas, insular, medial- and orbitofrontal areas and perirhinal cortex. These areas are likely more involved in processing of object information, attention and motivation. Third, we will compare the intrinsic networks involving principal- and inter-neurons in LEC and MEC. Together, these observations suggest that the different phenotypes of both EC subdivisions likely depend on the combination of intrinsic organization and specific sets of inputs. We further suggest a reappraisal of the notion of EC as a layered input-output structure for the hippocampal formation.

## Introduction

The denomination “entorhinal cortex (EC)” (Brodman’s area 28) is based on the fact that it is (partially) enclosed by the rhinal (olfactory) sulcus. Interest in the EC arose around the turn of the 20th century when Ramón y Cajal, described a peculiar part of the posterior temporal cortex that was strongly connected to the hippocampus by way of the temporo-ammonic tract (Ramón Y Cajal, 1902; Witter et al., in press). Cajal was struck by this massive connection and he therefore suggested that the functional significance of the hippocampus had to be related to that of EC or the sphenoidal cortex/angular ganglion, as he called it at that time. Today, EC is conceived as the nodal point between the hippocampal formation on the one hand and a variety of cortical areas on the other hand. Multimodal, as well as highly processed unimodal sensory inputs converge at the level of neurons in the superficial layers of the EC. This input is conveyed by the neurons in layers II and III of EC to all subdivisions of the hippocampal formation (Insausti et al., 2004; van Strien et al., 2009; Cappaert et al., 2014; Strange et al., 2014). The hippocampal fields CA1 and subiculum are the main source of projections that return to layer V of EC, with a less dense projection to layers II and III. Layer V neurons in turn are the main origin of EC projections to widespread cortical and subcortical domains in the forebrain (Rosene and Van Hoesen, 1977; Kosel et al., 1982; Cappaert et al., 2014).

EC comprises different subdivisions, charaterized by connectivity with functionally different sets of cortical and subcortical areas in the brain. This has led to the now quite widely accepted concept of parallel input/output channels, mediated by way of perirhinal and postrhinal (rodents) or parahippocampal cortex (primates; Witter et al., 1989a, 2000; Naber et al., 1997; Eichenbaum et al., 2012; Ranganath and Ritchey, 2012). Recent electrophysiological recordings in the lateral and medial EC (LEC and MEC respectively; see below for definitions) of rodents show that cells in MEC are predominantly spatially modulated. In contrast, in LEC such modulation is essentially absent, with neuron-firing correlating to objects in context (Fyhn et al., 2004; Deshmukh and Knierim, 2011; Knierim et al., 2013; Tsao et al., 2013; Moser et al., 2014). Does this phenotypical difference between the two EC components reflect input differences, or differences in local circuits and cell types, or could this phenotypical separation be the result of interactions between these two parameters. In this review, we aim to address specifically this question by providing a comprehensive description of EC, its intrinsic organization in relation to input and output organizations. We mainly focus on data from studies in rodents, although occasional comparative remarks are inserted when considered relevant for the narrative of the article.

## Definition of The Entorhinal Cortex, Subdivisions and Overall Architecture

There are different ways to define a cortical area, using different criteria, such as location, connectivity, cyto- and chemoarchitecture. Applying all of these approaches has resulted in a variety of borders, subdivisions and description of layers. Architectural parcellation schemes are useful tools to relate experimental data to standard locations in the brain (Bjaalie, 2002; van Strien et al., 2009; Zilles and Amunts, 2010; Kjonigsen et al., 2011, 2015; Boccara et al., 2015). Connection-based subdivision schemes may relate closer to our understanding of functional differences between areas (see below). In view of the strong implications of the human EC in a variety of brain diseases (Braak and Braak, 1992), the development of adequate animal models for such diseases depends strongly on our capabilities to extrapolate the definition of the EC from rodents to non-human and human primates. Therefore, combinations of the different approaches mentioned above will likely provide the most reliable concept for subdividing EC.

An apparently good lead, since it has withstood over a century of arguments, is the definition of EC based on hippocampal connectivity, as originally suggested by Ramón Y Cajal (1902, 1911). In view of increasing insights into the connectivity of the hippocampal formation and its subdivisions, we follow the well-established practice in rodents to take the differential distribution of EC projections to the dentate gyrus as a good defining criterion for two main subdivisions of EC. These are nowadays referred to as LEC and MEC (Steward, 1976; Witter, 2007). Unfortunately, in the monkey, the terminal distribution of the entorhinal-to-dentate projection does not provide such a clear criterion to functionally subdivide EC (Witter et al., 1989b). Potentially in line with this, cytoarchitectural division schemes tend to differentiate more than two subdivisions (Amaral et al., 1987; Rosene and Van Hoesen, 1987). However, the second entorhinal-hippocampal projection, connecting the two entorhinal domains to area CA1 and the subiculum in all mammalian species studied, including primates, shows a strikingly preserved topology along the transverse axis of both hippocampal fields. Projections emerging from a posteromedial location in EC target the proximal CA1, i.e., close to DG, and distal subiculum, whereas an anterolateral origin in EC maps onto the distal CA1 and adjacent proximal subiculum (human: Witter et al., 2000; Maass et al., 2015; monkey: Witter and Amaral, 1991; rat: Naber et al., 2001; van Strien et al., 2009).

Other connectivity patterns have been proposed to functionally subdivide EC as well, one being the input from the presubiculum. In all non-primate mammalian species studied so far, including rat, guinea pig and cat, the innervation of EC by presubicular fibers is restricted to a more caudal and dorsal portion that coincides with a cyto- and chemoarchitectonically well defined area, now called MEC (Shipley, 1975; Köhler, 1984; Room and Groenewegen, 1986). Also in the monkey, inputs from the presubiculum distribute to only a restricted posterior portion of EC (Amaral et al., 1984; Saunders and Rosene, 1988; Witter and Amaral, unpublished observations), and this area may thus represent the homolog of MEC as defined in non-primates. Recent connectional MRI studies in humans have pointed to a comparable connectional bipartite system separating anterolateral from posteromedial EC, showing clear differences with respect to connectivity measures with perirhinal and parahippocampal cortex, resembling those reported in rodents (Naber et al., 1997; Maass et al., 2015; Navarro Schröder et al., 2015).

Cytoarchitectural data reveal that in all species studied, two entorhinal areas can be differentiated and that these share cytoarchitectonic features with the two entorhinal areas defined by Brodmann as areas 28a and b (Brodmann, 1909). One can easily recognize a posteromedial area characterized by a very regular six-layered structure and a homogenous distribution of neurons in all layers, typical for area 28b or MEC. Layer II of MEC comprises a mixture of excitatory medium-sized pyramidal neurons and large multipolar neurons that have become known as stellate cells (SCs). On the opposite, anterolateral side, the laminar structure is comparable, but much less regular, resembling the cytoarchitecture of area 28a or LEC. In the latter portion, layer II comprises a mixture of large multipolar neurons, nowadays in rodents referred to as fan cells, pyramidal and medium-sized multipolar neurons. At some locations, these cell types seem to cluster into sublayers (referred to as IIa and IIb, or II and IIIa; Kobro-Flatmoen and Witter, 2017). Depending on the species, one or several additional subdivisions have been described, similar to what was mentioned above for the monkey (Lorente de Nó, 1933; Insausti et al., 1997). Note that the terms LEC and MEC do not simply reflect a particular position in anatomical or stereotaxic space. In many species, the two areas, defined by their combined architectural and hodological features occupy a more rostrolateral (LEC) vs. a more caudomedial position (MEC).

## Connectivity of The Two Entorhinal Subdivisions

Both LEC and MEC project to the hippocampus, and the axons form synapses on neurons in all hippocampal subfields. Neurons in layer II are the main source of the entorhinal projections to the dentate gyrus and fields CA2 and CA3, and neurons in layer III give rise to the entorhinal projections to CA1 and subiculum (note that a small number of neurons in deeper entorhinal layers contribute to both projections). In view of a confusing nomenclature that has developed over the years to describe these different projection systems (for a recent description and discussion, see Witter et al., in press), in the present article, we differentiate between the EC-layer II projection and the EC-layer III projection. Regarding the EC-layer II projection, we know that single layer II cells project to both the dentate gyrus and CA2/CA3 (Tamamaki and Nojyo, 1993). Whether such a collateral organization is true for the layer III projection to CA1 and subiculum is unclear. In view of this striking layer-separation in the origin of the EC to hippocampus projections, we feel that a description of intrinsic and extrinsic connectivity of LEC and MEC might benefit from a layered approach. In the following, we focus on the main cell layers II, III and V (for a description of layers I and VI, the reader is referred to Canto et al., 2008; Cappaert et al., 2014).

### Extrinsic Connections

The two entorhinal divisions differ with respect to their major extrinsic cortical and subcortical connections (for recent detailed overviews in the rat, see Kerr et al., 2007; Cappaert et al., 2014; for broader comparative overviews of cortical connectivity in a functional context, see Eichenbaum et al., 2012; Ranganath and Ritchey, 2012). Here we focus on a description of the distribution of main cortical inputs and their laminar preference of termination. Superficial layers of EC receive a substantial input from olfactory structures including the olfactory bulb, the anterior olfactory nucleus, and the piriform cortex (Haberly and Price, 1978; Kosel et al., 1981). Olfactory axons preferentially terminate laterally and centrally in LEC and in MEC, avoiding the most caudodorsal portion of MEC (Kerr et al., 2007). Olfactory fibers mainly distribute to layer I, where they make synaptic contacts with dendrites of neurons in layers II and III (Wouterlood and Nederlof, 1983). Other superficially terminating inputs to dorsolateral parts of LEC originate from insular cortex (Mathiasen et al., 2015), perirhinal cortex (Naber et al., 1999; Pinto et al., 2006) and orbitofrontal cortex (Hoover and Vertes, 2007, 2011; Kondo and Witter, 2014). Interestingly, the orbitofrontal and insular projections to LEC mainly terminate anteriorly, and close to the rhinal fissure. Parietal cortex projects moderately to LEC and MEC, terminating close to the rhinal fissure, preferentially in layers I and V (Olsen et al., 2017). Superficial layers of MEC receive inputs from the orbitofrontal cortex, but only from the ventral part (Kondo and Witter, 2014), postrhinal cortex (Koganezawa et al., 2015) and pre- and parasubiculum (Caballero-Bleda and Witter, 1993). The latter two inputs not only terminate on dendrites of neurons in layers II and III, but also influence neurons in layer V (Canto et al., 2012), and such a connectional scheme might hold true for all superficially terminating inputs. This however remains to be established, but the possibility points to a potentially relevant role for layer V neurons as integrators of entorhinal inputs, since they also are the recipients of other major cortical inputs distributing to layer V. These include inputs from infralimbic and prelimbic cortex, apparently innervating LEC and MEC almost equally dense. LEC layer V receives a denser input from anterior cingulate cortex, whereas the retrosplenial innervation almost exclusively distributes to MEC layer V (Wyss and Van Groen, 1992; Vertes, 2004; Jones and Witter, 2007), which also receives a weak to moderate input from visual cortex (Kerr et al., 2007; Olsen et al., 2017).

### Intrinsic Networks Layer II

**Figure 1:**
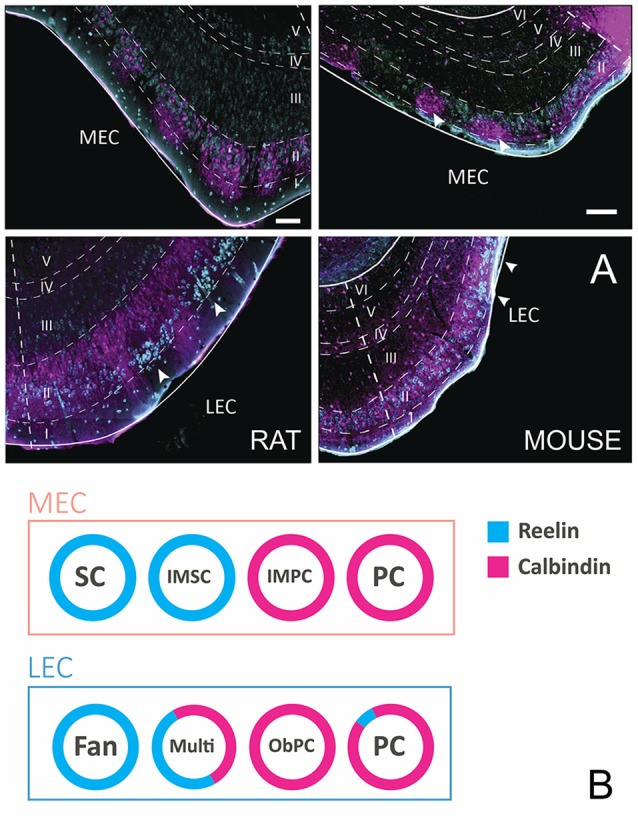
Layer II cells come in two, chemically defined types, reelin- and calbindin-positive. **(A)** Coronal sections taken through entorhinal cortex (EC) of the rat (left) and mouse (right), stained for reelin (cyan) and calbindin (magenta). Note the different position of the two cell populations in the two species and the two subdivisions of EC. In the rat medial EC (MEC; top-left), the two populations are intermingled with a tendency for both types to cluster somewhat. In contrast, in mouse MEC (top-right), calbindin-positive cells form clear clusters (white arrowheads) that are located superficial to the reelin-positive neurons. In lateral EC (LEC) of the rat (bottom-left), reelin-positive cells form superficially positioned clusters (white arrowheads), separated by calbindin-positive dendritic bundles belonging to the deeper positioned, equally dispersed calbindin-positive neurons. In LEC of the mouse (bottom-right), a more equal distribution is seen, although two superficially located reelin clusters are present (white arrowheads). Scale bars equal 100 μm. **(B)** Schematic representation of the relationships of morphologically, electrophysiologically and connectionally defined cell types, and their chemical phenotype in LEC and MEC. Abbreviations: Fan, fan cell; IMSC, intermediate stellate cell; IMPC, intermediate pyramidal cell; multi, multipolar cell; ObPC, oblique pyramidal cell; PC, pyramidal cell; SC, stellate cell.

Principal cells in both subdivisions of EC come in two chemical types, calbindin- and reelin-expressing cells. In MEC, calbindin-positive cells and reelin-positive cells appear to be grouped in patches, and in LEC the two cell types are more or less confined to two separate sublayers, reelin cells in layer IIa and calbindin cells in layer IIb. The reported clustering of calbindin-positive neurons is particularly striking in limited parts of MEC and is more striking in mice than in rats or other species. Only in mouse MEC the calbindin-positive neurons are located superficial to the reelin positive neurons (Figure 1A; Tunon et al., 1992; Fujimaru and Kosaka, 1996; Wouterlood, 2002; Ramos-Moreno et al., 2006; Kitamura et al., 2014; Ray et al., 2014; Leitner et al., 2016). EC in humans is known for its wart-like bumps or verrucae (Retzius, 1896; Klinger, 1948; Solodkin and Vanhoesen, 1996; Naumann et al., 2016), which in the largest part of EC, located centrally along the anteroposterior and lateromedial axes, are composed of the large multipolar reelin positive layer II cells, described as the pre-alfa neurons by Braak (Braak and Braak, 1985; Tunon et al., 1992; Kobro-Flatmoen et al., 2016; Naumann et al., 2016). Moreover, the marked clustering of calbindin-positive neurons in all species studied is limited to a restricted posterior part of MEC (Naumann et al., 2016). In our view, it is therefore confusing to refer to calbindin-positive cells in layer II as island cells embedded in an ocean of reelin-positive cells (Kitamura et al., 2014), since this organization is likely opposite for the larger part of EC. Reelin-positive cells in both entorhinal areas project to the dentate gyrus and CA3, whereas calbindin-positive neurons project to several other targets including the CA1 and the contralateral EC, the olfactory bulb and piriform cortex (Varga et al., 2010; Kitamura et al., 2014; Fuchs et al., 2016; Leitner et al., 2016; Ohara et al., 2016). The two chemically defined cell groups are composed of several morphological subgroups that can be distinguished based on somatic and dendritic features (Canto and Witter, 2012a,b; Fuchs et al., 2016; Leitner et al., 2016).

In MEC, SCs make up the largest subgroup of principal cells. They have multiple primary dendrites that radiate out from a round soma. SCs are typically reelin-positive and calbindin-negative. Medium to large pyramidal cells (PCs) make up the other main principal cell type in layer II of the MEC. PCs are typically calbindin-positive, although a few reelin-positive PC have been described (Fuchs et al., 2016; Figure 1B). There are at least two intermediate cell groups in between stellate and pyramidal morphologies, here referred to as intermediate SCs (IMSCs) and intermediate PCs (IMPCs). IMSCs all express reelin, but a few of them co-express calbindin, the IMPCs tend to be calbindin-positive, but are more diverse and come in both reelin-positive and reelin and calbindin co-expressing varieties. The four principal cell types in the MEC can also be distinguished from each other based on their electrophysiological profiles (Canto and Witter, 2012b; Fuchs et al., 2016).

In LEC layer II, there are also at least four subgroups of principal cells (Canto and Witter, 2012a; Leitner et al., 2016). Fan cells are similar in morphology to SCs, but lack a distinctive basal dendritic tree (Tahvildari and Alonso, 2005; Canto and Witter, 2012a). Most are reelin-positive, though some may express calbindin. PCs make up the other large group of principal cells in LEC, they are morphologically similar to those described in MEC. They are largely calbindin-positive, but some may be reelin-positive. Oblique PCs (ObPCs) and multipolar cell make up the intermediate cell types in the LEC (Canto and Witter, 2012a; Leitner et al., 2016). Oblique pyramidals display a morphology similar to PCs, but are tilted relative to the pial surface, and they predominantly express calbindin. Multipolar cells, on the other hand, have a more diverse morphology, and express both calbindin and reelin (Figure 1B). Electrophysiologically, the four cell groups in LEC are not as easily distinguishable as in MEC, however recent data suggest that there may be subtle physiological differences between the overarching reelin and calbindin classes (Tahvildari and Alonso, 2005; Canto and Witter, 2012a; Leitner et al., 2016).

Similar to what has been reported for neocortical areas, EC has been suggested to contain three main subgroups of interneurons, parvalbumin (PV), somatostatin (SOM) and 5HT3a expressing cells (Rudy et al., 2011; Fuchs et al., 2016; Leitner et al., 2016). PV-positive interneurons constitute approximately half of the interneuron population across EC, making them the largest subgroup of interneurons in the area (Wouterlood et al., 1995; Miettinen et al., 1996). Layer II of MEC has a large number of PV expressing somata and heavy neuropil staining. Layer II of LEC has comparatively weak PV staining, with few somata and light neuropil staining. Particularly layer IIa appears to lack PV-positive cells (Wouterlood et al., 1995; Fujimaru and Kosaka, 1996; Miettinen et al., 1996; Leitner et al., 2016). In both LEC and MEC, there is a clear gradient of PV staining, with portions close to the rhinal fissure expressing more than ventral portions (Wouterlood et al., 1995; Fujimaru and Kosaka, 1996; Leitner et al., 2016). A comparable, and strikingly strong gradient has been reported in relation to the collateral and rhinal sulcus in primates (human: Tunon et al., 1992; monkey: Pitkanen and Amaral, 1993; for a detailed comparative description, see Kobro-Flatmoen and Witter, 2017).

Like PV cells in other parts of the brain (Hu et al., 2014), those in layer II of MEC are known to display a fast spiking physiological profile (Couey et al., 2013; Pastoll et al., 2013; Armstrong et al., 2016; Fuchs et al., 2016; Leitner et al., 2016). The existence of PV-positive baskets surrounding principal cells in layer II is supported by both histological and electrophysiological studies (Jones and Bühl, 1993; Wouterlood et al., 1995; Varga et al., 2010; Armstrong et al., 2016; Fuchs et al., 2016). Another type of basket cell in layer II of MEC is the CCK-expressing basket cell (Varga et al., 2010; Armstrong et al., 2016). These cells are less abundant than PV-expressing cells, and constitute a subgroup of the 5HT3aR expressing interneurons (Lee et al., 2010). Whereas CCk-positive basket cells preferentially target calbindin-positive principal cells, single PV-positive basket cells innervate both reelin- and calbindin-positive neurons (Armstrong et al., 2016). Basket cells have also been described in layer II of the LEC, but no details are available about different types and abundance, nor how they are part of the LEC microcircuit.

A second, common type of GABAergic interneuron that expresses PV in layer II, also present in layer III, is the chandelier or axo-axonic cell. Chandelier cells are characterized by vertical aggregations of axonal boutons, called candles which mainly make synapses on the initial axon segments of principal cells. In MEC, both vertical and horizontal chandelier cells are present, and in LEC the horizontal subtype is dominant. The local axon branches of these neurons are largely confined to layers II and III (Soriano et al., 1993).

**Figure 2:**
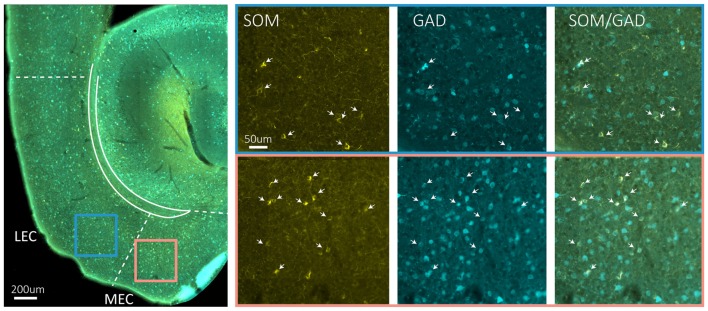
Somatostatin neurons are GAD67 positive. The left hand side main panel shows a low power image of a horizontal section obtained from a GAD67 transgenic line expressing GFP (Tanaka et al., 2003), stained for the expression of somatostatin. The colored squares indicate the position of the high power images shown on the right. Blue square is LEC, red square is MEC. The solid blueish staining at the edge of EC is an artifact due to overlying cerebellar tissue. On the right hand side, high power images show the indicated areas in LEC and MEC in three different fluorescent channels from left to right: somatostatin (yellow), GFP (cyan) and overlay of somatostatin and GFP. Scale bars equal 200 μm in the left main panel and 50 μm for the six panels on the right-hand side.

Immunohistochemical studies describing the distribution of somatostatin expressing somata in EC are conflicting, particularly with regards to distribution in superficial layers. However, no major differences between entorhinal subdivisions have been described (Köhler and Chan-Palay, 1983; Wouterlood and Pothuizen, 2000). Somatostatin cells in MEC are generally multipolar low threshold spiking neurons (Couey et al., 2013; Fuchs et al., 2016). Available data indicate that only a small percentage of somatostatin neurons in EC are GABAergic (Wouterlood and Pothuizen, 2000), but our own data in mice show that most somatostatin neurons in EC are GABAergic (Figure 2). The last major interneuron group in EC, the 5HT3aR cells, consist of several subgroups, including calretinin-, VIP- and CCK-expressing cells (Lee et al., 2010; Fuchs et al., 2016; Leitner et al., 2016). 5HT3aR cells in layer II of MEC have diverse morphological and physiological profiles (Canto et al., 2008; Fuchs et al., 2016).

**Figure 3:**
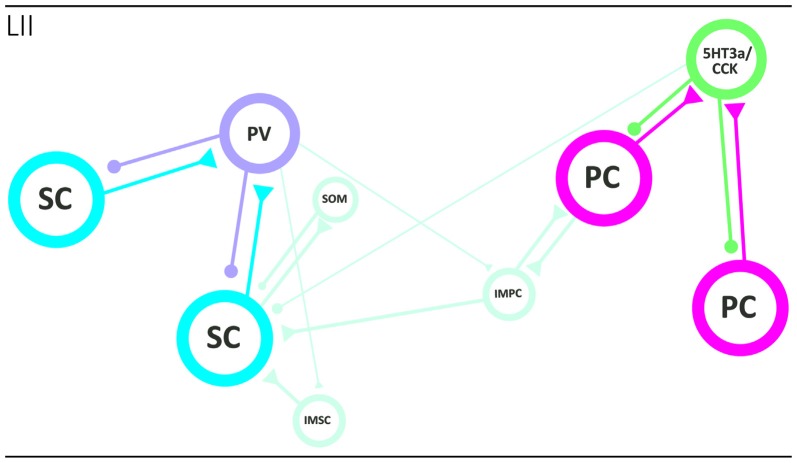
Local circuits in layer II of MEC. The two main principal neurons, stellate (cyan; left side) and pyramidal (magenta; right side) cells form two specific networks. SCs are not monosynaptically connected and that seems true for PCs as well. They are disynaptically connected preferentially by way of different interneuron subtypes, the fast-spiking PV positive basket cell in case of SCs and the 5HT3a/CCK-type in case of pyramidal neurons. The two networks are likely interconnected by way of intermediate pyramidal neurons (light blue), and PV interneurons may also target intermediate pyramidal and SCs. See text for further details. Abbreviations: IMSC, intermediate stellate cell; IMPC, intermediate pyramidal cell; PC, pyramidal cell; PV, parvalbumin expressing fast spiking basket cell; SC, stellate cell; SOM, somatostatin-expressing interneuron; 5HT3a/CCK, basket cell that expresses CCK and likely belongs to larger group of interneurons that express the 5HT3a receptor.

The regular grid pattern, typically seen in layer II of MEC has been hypothesized to emerge from the structure of microcircuits within layer II (Fuhs and Touretzky, 2006; McNaughton et al., 2006; Burak and Fiete, 2009; Bonnevie et al., 2013; Couey et al., 2013). The majority of grid cells in MEC are observed in layer II (Hafting et al., 2005; Sargolini et al., 2006), and the anatomical correlates of grid cells likely comprise both stellate-like and pyramidal-like cells (Domnisoru et al., 2013; Schmidt-Hieber and Häusser, 2013; Tang et al., 2014). The local circuit of SCs has been probed in several studies using *in vitro* patch clamp recordings, and it is now well established that individual SCs do not form monosynaptic connections with other SCs. Communication between SCs occurs through an intermediate inhibitory interneuron, in a mechanism by which activation of one or more SCs evokes disynaptic inhibitory currents in neighboring SCs. Paired recordings have revealed strong connectivity in both directions between SCs and fast-spiking cells and, to a much lesser extent, between SCs and low-threshold spiking interneurons (Couey et al., 2013; Pastoll et al., 2013; Fuchs et al., 2016). The functional disynaptic link that illustrates the core principle of the stellate microcircuit is mediated by a single type of inhibitory neuron, the PV positive fast spiking cell (Figure 3; Buetfering et al., 2014; Armstrong et al., 2016).

The local network of PCs has been explored using similar methods, and like the SC network, very sparse monosynaptic connectivity was detected between PCs. These results suggest that the general principle of disynaptic connectivity as described for the SC network also applies to the layer II PCs. An important distinction however is that PCs seem to communicate through different subsets of interneurons. In contrast to SCs, PCs are not connected, in either direction, to PV positive fast-spiking cells or somatostatin positive low threshold spiking cells, but instead form synaptic connections solely with the heterogeneous 5HT_3A_ expressing population of interneurons (Figure 3; Fuchs et al., 2016).

Synaptic interaction between the pyramidal and SC networks is limited, as available data points to little monosynaptic connectivity between stellate and PCs (Couey et al., 2013; Fuchs et al., 2016). This suggest the existence of two isolated subcircuits within layer II of MEC, where information relayed to the dentate gyrus by reelin positive SCs is processed separately from information relayed by calbindin positive PCs to other downstream areas. However, it should be kept in mind that the networks may be coordinated through one of the intermediate cell types, e.g., the IMPCs, which have been shown to form synaptic connections with both pyramidal and SCs (Figure 3; Fuchs et al., 2016).

If the local microcircuit design of layer II MEC excitatory cells is crucial for generating grid cell firing, the absence of grid cells in LEC predicts a different organization of the layer II principal cell microcircuit. Given the observation that inhibition dominates microcircuits of both pyramidal and SCs in MEC, albeit provided by different types of interneurons, comparable cell types in the LEC, e.g., the fan and PC, may have a circuit structure where monosynaptic connectivity prevails. Our preliminary data from paired recordings of fan cells indicates that direct communication between cells of this type is present, but not prevalent (Nilssen et al., 2015). Potential microcircuit differences between layer II of MEC and LEC might also reflect different contributions from the local interneuron population. In LEC, 5HT3aR expressing interneurons constitute the largest interneuron group in layer II, unlike in the MEC, where PV cells are thought to be the predominant interneuron group (Leitner et al., 2016). This finding indicates that the inhibitory systems in MEC and LEC layer II are dominated by different subtypes of interneurons.

### Layer III

Compared with what is known about neurons and connectivity in layers II and V, Layer III is still largely terra incognita. Layer III in both LEC and MEC comprises a homogenous population of spiny excitatory pyramidal neurons that project to CA1 and subiculum (Tahvildari and Alonso, 2005; Canto and Witter, 2012a,b; Tang et al., 2015). Layer III neurons also project contralaterally to the hippocampus and EC (Steward and Scoville, 1976). About 40% of the layer III hippocampal projecting cells in MEC send collaterals to the contralateral MEC (Tang et al., 2015). The axons of the commissural projecting cells in MEC apparently distribute mainly to layer III, thus contrasting to the small percentage of commissural calbindin-positive neurons in layer II, of which the axons preferentially distribute in layer I of the contralateral MEC (Fuchs et al., 2016). In addition, layer III also contains a population of non-spiny PCs, sending axons towards the angular bundle. Collaterals originate from the main axon close to the cell body and those traveling towards the superficial layers distribute over the own dendritic extent (Gloveli et al., 1997). The third principal neuron type in layer III is formed by multipolar neurons. These contribute to the hippocampal projections (Germroth et al., 1989). Layer III contains a variety of interneurons, exhibiting various morphologies, including multipolar, pyramidal and bipolar neurons. Chemical characterization of layer III interneurons in the MEC shows that they express several markers including somatastatin, calbindin, vasoactive intestinal peptide and substance-P (Köhler and Chan-Palay, 1983; Köhler et al., 1985; Gloveli et al., 1997; Wouterlood and Pothuizen, 2000; Wouterlood et al., 2000; Kumar and Buckmaster, 2006).

The microcircuits of layer III are only sparsely known, but seem to be markedly different from those seen in layer II, showing a much stronger monosynaptic principal to principal neuron connectivity (van der Linden and Lopes da Silva, 1998; Dhillon and Jones, 2000; Kloosterman et al., 2003; Tang et al., 2015). Neurons in layer III are the main recipients of the local deep-to-superficial projections, which apparently predominantly originate from neurons in layer Vb (see below; Kloosterman et al., 2003; van Haeften et al., 2003). Currently, no correlations have been reported between morphology, connectional profile and electrophysiological *in vitro* and *in vivo* properties (Canto and Witter, 2012a,b; Tang et al., 2015).

### Layer V

As described above, layer V is commonly subdivided into a layer Va and Vb. The superficial layer Va, adjacent to layer IV (lamina dissecans), comprises mainly large pyramidal neurons that are unequally distributed along the extent of both MEC and LEC. Cells in layer Vb appear smaller, more uniform in soma size and are more densely packed than their counterparts in layer Va (Canto and Witter, 2012a,b; Boccara et al., 2015).

**Figure 4:**
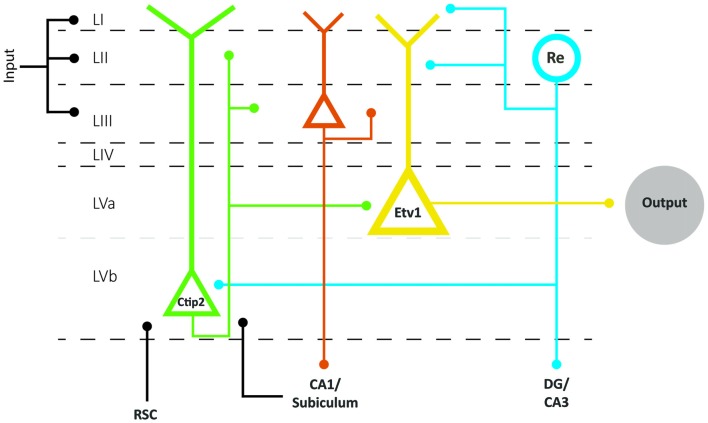
Schematic representation of the layer V network as part of the EC network. Layer V comprises two sublayers Va and Vb, based on the differential expression of two transcription factors, Etv1 and Ctip 2, respectively. Layer Va neurons are the main origin of projections to cortical and subcortical structures in the brain. Layer Vb neurons receive inputs from the hippocampus and RSC and project locally to Va and superficial layers. Superficial inputs likely form synapses onto dendrites of principal neurons in layers II, III and V of EC. Neurons in layer II and III provide the main input to the hippocampus, which is returned to layer VB and subsequently made available to layer Va neurons, which originate the main outbound projections of EC. Neurons in layer Vb are also the main source of back projections to layer II and III neurons. The scheme clearly shows that we lack detailed connectional data on layer III as well as on input specificity to layer Va and Vb neurons. Abbreviations: Re, reelin-expressing neurons; RSC, retrosplenial cortex.

In mice, the expression pattern of the transcription factors Etv1 and Ctip2 provide for the differentiation between two molecularly distinct sublayers Va and Vb, respectively. This organization prevails across the whole mediolateral and dorsoventral extent of EC (Ramsden et al., 2015; Surmeli et al., 2015; Onodera et al., 2016). In both MEC and LEC, layer Va cells are the major output neurons projecting to diverse cortical and subcortical structures. Surprisingly, layer Vb cells are selectively targeted by the outputs from the hippocampus, originating in CA1 and subiculum as well as by projections originating in layer II of EC (Figure 4; Surmeli et al., 2015; Onodera et al., 2016). In MEC, these layer II inputs apparently arise specifically from reelin positive MEC II SCs and not from the calbindin positive MEC II PCs (Surmeli et al., 2015). The latter report of axon collaterals from layer II SCs in layer V in mice conflicts with previous reports in rats and monkeys, that layer II SCs issue a well-developed axonal plexus in layers I and II, but that collaterals in layer V are sparse (Tamamaki and Nojyo, 1993; Klink and Alonso, 1997; Buckmaster et al., 2004; Canto and Witter, 2012b). Whether this points to species differences or a lack of sensitivity in the older studies is not known. Irrespective of the details of this circuit, MEC layer Vb neurons could be ideally suited to integrate inputs from superficial MEC and hippocampus. Own preliminary data show these network features to be true in LEC as well, and show that layer Vb neurons in both LEC and MEC innervate layer Va as well as layers II and III (Onodera et al., 2016), which is in line with sparse data indicating that neurons in layer Vb issue superficially directed axon collaterals (Hamam et al., 2000, 2002; Canto and Witter, 2012a,b). This indicates that at least a subpopulation of layer Vb neurons form a major component of the intrinsic deep to superficial circuit.

Layer V is also innervated by additional cortical projections from frontal and cingular domains (see above). Whereas information about the postsynaptic targets of these cortical inputs is sparse, projections from the retrosplenial cortex (RSC) to MEC layer V target, among others, spiny pyramidal neurons that issue axons to superficial layers (Czajkowski et al., 2013). If the assertion is correct that in particular layer Vb neurons are the main elements mediating this deep to superficial connection, it is logical to conclude that retrosplenial inputs terminate onto a subpopulation of Vb neurons (Figure 4). These data are thus in line with own preliminary observations that neurons in layer V receive convergent inputs from subiculum and RSC (Simonsen et al., 2012).

Layer Vb of both MEC and LEC also contains multipolar neurons (Hamam et al., 2000; Canto and Witter, 2012b) and a population of GABA-negative/calretinin positive neurons (Miettinen et al., 1997) providing additional markers for principal cell types in the layer V network. Electrophysiologically, PCs in layer V show regular spiking, strongly adapting physiological profiles, whereas multipolar neurons respond to a depolarization with delayed firing and slow little adaptation (Egorov et al., 2002b). It is currently not known if any of these layer V cell types correlate with the electrophysiologically defined persistent firing neurons, which can be found in EC when muscarinic acetylcholine receptors are activated (Egorov et al., 2002a). Finally, we currently lack a detailed comparison of the organization of layer V in LEC and MEC. For example, what would be the functional implication that MEC layer Va hosts pyramidal neurons with extensive basal dendritic trees restricted to the somatic layer, whereas such a neuron type has not been reported in LEC (Hamam et al., 2000, 2002; Canto and Witter, 2012a,b; Surmeli et al., 2015).

## Concluding Remarks

The comparison of main trends in extrinsic and intrinsic connectivity patterns of MEC and LEC suggests that the different phenotypes of both EC subdivisions likely depend on the combinatorial effects of small differences in intrinsic organization and substantial differences in extrinsic inputs. Although this conclusion and the following details are mainly based on studies in rodents, the more sparse data in non-human and human primates seem to support a comparable organization. To understand the functional relevance of the subtle intrinsic differences, more data are needed, for which we likely will depend on the emergence of even more specific genetic tools to identify and manipulate the activity of single classes of neurons. Eventually, detailed imaging studies in humans are expected to contribute to an increased understanding of the functional diversification within EC. The extrinsic input differences as summarized above are still in overall support with the notion that two functionally different input streams to the hippocampus are mediated by two entorhinal domains. MEC provides connectional routes with extensive posterior parts of the cortex, including posterior parahippocampal, retrosplenial, parietal and occipital networks, allowing the representation of intrinsically generated signals about perceived and/or planned movements in stable contexts. In contrast, LEC mediates routes to and from the hippocampus with more anterior parahippocampal, sensory and pre- and orbitofrontal domains, providing access to evaluated information about the ever-changing external world. From a functional anatomical perspective, the above provides a suitable framework to keep adding the details needed to mechanistically understand the role(s) of EC. The connectional scheme as presented here (Figure 4) assumes that the functionally different parts of EC share the network structure to mediate cortical-hippocampal interactions in a comparable matter. Neurons in layers II and III provide various combinations of information to the hippocampal circuit, and a copy of that input is made available to neurons in layer V. The latter step might either be monosynaptic through inputs targeting the extensive apical tufts of some of the layer V pyramidal neurons or disynaptic through intrinsic projections from layer II (and layer III) to layer Vb. In view of the strict topology of the reciprocal connectivity between EC and CA1/subiculum, it is likely that at least some of these layer Vb neurons receive a hippocampally processed copy of that original input information. Layer Vb neurons are in a position to integrate those inputs with additional sets of information, and to send the resulting representations back to layers II and III. In case of layer Va neurons, which apparently are the origin of the main output pathway of EC, the hippocampally processed copy might be disynaptical, mediated through Vb neurons, and it is currently not known whether other inputs integrate at the level of these Va neurons. In view of their apical dendrites reaching the superficial layers of EC, it is likely that they, like layer Vb neurons, do receive superficially terminating inputs.

If correct, the connectional data strongly argue that differences in cortical inputs form a main feature underlying the phenotypic differences between LEC and MEC. However, we have not yet included the potential differences between LEC and MEC in local inhibitory architecture, as suggested by the yet sparse data on layer II. One additional feature of the proposed scheme needs to be discussed. The overarching strict reciprocal topology of the entorhinal-CA1-subicular network predicts that inbound information will be reciprocated with outbound information. It is exactly this last prediction, which is not supported by data. Admittedly, the available data are sparse, but the data obtained in the few studies in which this input-output dogma was addressed point to another direction. In one study in the cat, EEG recordings in freely behaving animals indicated a functional separation between LEC and MEC, where LEC is coupled to the olfactory domain, whereas MEC is coupled to the hippocampus (Boeijinga and Lopes da Silva, 1988). In more elaborate studies using the isolated guinea pig *ex vivo* brain preparation, olfactory stimulation resulted in a sequential activation in LEC, hippocampus and MEC, followed by LEC (Biella and de Curtis, 2000). These sparse data seem to indicate that hippocampal output, resulting from olfactory input, is preferentially distributed back to MEC, not to LEC. To our knowledge, this output pathway specificity has not been explored and thus presents us with a, yet underexplored, challenge, which might very well be open to imaging studies in the human.

## Author Contributions

All authors contributed to the discussions that formed the foundation of the manuscript and contributed to the writing of the manuscript and to figures. All figures with exception of 1A were made by BJ. MPW supervised the process and wrote the final version of the manuscript. All authors approved this final version.

## Conflict of Interest Statement

The authors declare that the research was conducted in the absence of any commercial or financial relationships that could be construed as a potential conflict of interest.
